# Antimicrobial Properties of a Novel PEGylated Copper Nanoparticle-Embedded Silicone Rubber with Potential for Use in Biomedical Applications

**DOI:** 10.3390/polym17101404

**Published:** 2025-05-20

**Authors:** Sara Ramírez Pastén, Carolina Paz Quezada, Carolina Arellano, Roberto M. Vidal, Alejandro Escobar, Faustino Alonso, Javier Villarroel, David A. Montero, María C. Paredes

**Affiliations:** 1 Centro Integrativo de Biología y Química Aplicada (CIBQA), Universidad Bernardo O’Higgins, Santiago 8370993, Chile; sara.ramirez@ubo.cl (S.R.P.); carolina.pqb@gmail.com (C.P.Q.); 2 Departamento de Química Ambiental, Facultad de Ciencias, Universidad Católica de la Santísima Concepción, Concepción 4070129, Chile; 3 Programa de Microbiología y Micología, Instituto de Ciencias Biomédicas, Facultad de Medicina, Universidad de Chile, Santiago 8380453, Chile; caroarellanocabezas@gmail.com (C.A.); rvidal@uchile.cl (R.M.V.); 4 Instituto de Investigación en Ciencias Odontológicas, Facultad de Odontología, Universidad de Chile, Santiago 8380544, Chile; janodvm@gmail.com; 5 Instituto de Salud Poblacional, Facultad de Medicina, Universidad de Chile, Santiago 8380453, Chile; falonso@uchile.cl; 6 Unidad de Infectología, Servicio de Medicina, Hospital del Salvador, Servicio de Salud Metropolitano Oriente, Santiago 7500922, Chile; jvillarroel@hsalvador.cl; 7 Carrera de Enfermería, Facultad de Salud, Sede Santiago, Universidad Santo Tomás, Santiago 8370003, Chile

**Keywords:** antimicrobial polymers, antibiofilm, biofilm catheter-associated pathogens

## Abstract

**Background**: Healthcare-associated infections (HAIs) significantly increase morbidity, mortality, and healthcare costs. Among HAIs, catheter-associated infections are particularly prevalent due to the susceptibility of catheters to microbial contamination and biofilm formation, especially with prolonged use. Biofilms act as infection reservoirs, complicating treatment and often requiring catheter removal, thus extending hospital stays and increasing costs. Recent technological advances in catheter design have focused on integrating antifouling and antimicrobial coatings to mitigate or prevent biofilm formation. **Methods**: We developed COPESIL^®^, a novel silicone rubber embedded with PEGylated copper nanoparticles designed to reduce microbial contamination on catheter surfaces. We conducted *in vitro* assays to evaluate the antimicrobial and antibiofilm efficacy of COPESIL^®^ against pathogens commonly implicated in catheter-associated urinary tract infections. Additionally, the safety profile of the material was assessed through cytotoxicity evaluations using HepG2 cells. **Results**: COPESIL^®^ demonstrated substantial antimicrobial activity, reducing contamination with *Escherichia coli* and *Klebsiella pneumoniae* by >99.9% and between 93.2% and 99.8%, respectively. Biofilm formation was reduced by 5.2- to 7.9-fold for *E. coli* and 2.7- to 2.8-fold for *K. pneumoniae* compared to controls. Cytotoxicity assays suggest the material is non-toxic, with cell viability remaining above 95% after 24 h of exposure. **Conclusions**: The integration of PEGylated copper nanoparticles into a silicone matrix in COPESIL^®^ represents a promising strategy to enhance the antimicrobial properties of catheters. Future studies should rigorously evaluate the long-term antimicrobial efficacy and clinical safety of COPESIL^®^-coated catheters, with a focus on their impact on patient outcomes and infection rates in clinical settings.

## 1. Introduction

Healthcare-associated infections (HAIs) pose a global health problem, significantly increasing morbidity, mortality, and medical costs. HAIs are primarily linked to invasive procedures, surgeries, prosthetic devices, and indwelling medical devices, such as catheters. Notably, catheter-related bloodstream infections (CRBSIs) and catheter-associated urinary tract infections (CAUTIs) rank among the most prevalent HAIs, underscoring the critical need for effective control measures [1,2,3,4].

Catheters are commonly fabricated from materials like polyurethane (PU), polyvinylchloride (PVC), latex, and polydimethylsiloxane (PDMS). Silicone stands out due to its biocompatibility, flexibility, and resistance to encrustation when exposed to bodily fluids, making it an ideal material for catheter manufacturing and other medical applications [5,6,7,8].

While clean and sterile techniques during catheter insertion significantly reduce the risks of catheter-associated infections [9,10], prolonged use may result in catheter contamination, either by microbiota or environmental microorganisms. Moreover, catheter contact with bodily fluids facilitates the adsorption of host-derived biomolecules such as collagen and fibronectin, thereby promoting microbial adhesion [11,12,13,14]. As a result, a biofilm may form on the catheter surface, which acts as a microbial reservoir for infection [15].

Treatment of CAUTIs involves antimicrobial therapy; however, the specific treatment is contingent upon the patient’s condition (symptomatic or asymptomatic), the type of catheter, and the microorganism responsible for the infection. Unfortunately, the resistance of biofilms to antimicrobial agents and host defenses complicates treatment strategies, potentially leading to severe outcomes such as sepsis and death [15,16,17]. Consequently, catheter removal often becomes imperative, extending hospitalizations and escalating medical costs [2,15,18].

Recent advances in catheter manufacturing include the integration of antifouling and antimicrobial coatings to inhibit biofilm formation. In particular, antifouling coatings are designed to prevent protein adsorption and microbial adhesion by modifying surface topography or chemistry [14]. Various polyhydrophilic and zwitterionic polymers have been used for this purpose. These polymers form a hydration layer on the material surface that acts as a physical and energetic barrier that prevents protein adsorption and is unfavorable for microbial attachment [19]. Among these, polyethylene glycol (PEG) is widely used due to its biocompatibility [13,20], with its antifouling mechanism attributed to the formation of a hydration layer and steric hindrance, which depends on the polymer chain length and surface packing density [21,22,23,24]. Despite their benefits, these coatings do not completely prevent microbial adhesion or eliminate microorganisms, eventually allowing biofilm formation [14].

The research into antimicrobial coatings for catheters has explored the use of antibiotics [8], antimicrobial peptides [25], and metals like silver, gold, platinum, zinc, and copper nanoparticles. In particular, copper nanoparticles (CuNPs) are notable for their affordability, broad-spectrum antimicrobial activity, and potential to control HAIs [2,26,27,28,29]. Although the exact mechanism is not fully understood, the antimicrobial activity of CuNPs is thought to involve disruption of microbial cell walls and membranes, induction of oxidative stress, and DNA damage [26,27,30,31,32,33]. However, CuNPs are sensitive to oxidation and tend to aggregate [26]. To stabilize them, they are often chemically functionalized with polymers like PEG [34] and others [32,35,36,37,38]. These capping agents improve the biocompatibility of CuNPs while maintaining their antimicrobial activity, making them more suitable for biomedical applications.

Despite promising *in vitro* results, antimicrobial catheters have shown inconsistent clinical outcomes in reducing biofilm formation and infection incidence [17,39,40]. This limited effectiveness may be due to insufficient prevention of microbial adhesion, as well as possible encrustation of catheter surfaces by host-derived molecules, which hinder the action of antimicrobial compounds [41,42]. Additionally, the leaching of antimicrobial agents over time may be a limiting factor [39,43]. These challenges highlight the need for more effective antibiofilm coatings with long-lasting antifouling and antimicrobial properties.

In this study, we developed a PEGylated copper nanoparticle-embedded poly(dimethylsiloxane) (COPESIL^®^) rubber with antimicrobial properties for use in biomedical applications, particularly aimed at reducing HAIs. This innovative material was formulated by anchoring poly(ethylene glycol)-containing silane (PEG-Silane) to the surface, providing antifouling properties. Subsequently, the as-prepared PEGylated silicone was coated with CuNPs that were previously stabilized with PEG, providing antimicrobial activity. COPESIL^®^ showed *in vitro* antimicrobial activity against common CAUTI-causing bacteria, including *Escherichia coli*, *Klebsiella pneumoniae*, *Enterococcus faecalis*, and *Pseudomonas aeruginosa*. More importantly, this material showed antibiofilm activity against *E. coli* and *K. pneumoniae.* Our findings aim to contribute to developing next-generation antibiofilm materials, providing an innovative solution to one of the most pressing problems in healthcare.

## 2. Materials and Methods

### 2.1. Silicone Films, Polyethylene Glycol, and Other Chemical Reagents

Commercial silicon membranes [poly(dimethylsiloxane), PDMS, 20 mm diameter, 0.024″ thickness] were provided by Interstate Specialty Products (Sutton, MA, USA). 2-[methoxypoly(ethyleneoxy)-6-9-propyl]trimethoxysilane (PEG-Silane) (Mw: 460–590; purity > 90%; actual chain length distribution as measured with GC/MS, silanes with 4–8 PEG units constitute 95% of the material) was provided by Gelest Inc., (Morrivilles, PA, USA). Copper (II) sulfate (CuSO_4_), sodium hydroxide (NaOH), l-ascorbic acid (AA), and polyethylene glycol (PEG) (Mw: 2000, 3000, 4000) were acquired from Sigma-Aldrich (Burlington, MA, USA). All the solutions were prepared using deionized water.

### 2.2. Formulation of COPESIL^®^

COPESIL^®^ represents an advanced technology designed to enhance the antimicrobial and antibiofilm capabilities of silicone materials. This technology involves a multi-step process of modifying, PEGylating, and functionalizing silicone surfaces. Key to this process is the anchoring of organic molecules that act as molecular wires, effectively transforming the surface properties of the silicone. Additionally, the technology incorporates the immobilization of CuNPs directly onto the silicone surface. These modifications confer anti-adhesive and antibiofilm properties designed to prevent microbial colonization of the material.

The chemical functionalization of the silicone surface was achieved through sequential silanization reactions to modify poly(dimethylsiloxane) substrate surfaces. CuNPs were PEG-grafted, PEGylated, and anchored by the molecules of [methoxy(polyethyleneoxy)propyl]trimethoxysilane (PEG-Silane) to the surface [44].

The CuNP suspension was obtained following the method previously reported with modification in the PEG length per chain [45]. We used PEGs of 2000, 3000, and 4000 Da (PEG2000, PEG3000, and PEG4000).

The concentration of the CuNP suspension was determined by the DLS technique of concentration in solution in the Nano tracking Analisys (NTA) equipment, model NanoSight N3 (Malvern Panalytical, Malvern, UK).

Two different dispersions were utilized: condition 1 (COPESIL-1; C1), where the volumes were multiplied by a factor of 5; and condition 2 (COPESIL-2; C2), where they were multiplied by a factor of 7.

The COPESIL^®^ samples were characterized using an HR-SEM Zeiss brand model EVO MA10 scanning electron microscope equipped with a tungsten filament, and combined with energy dispersive spectroscopy (EDS) with an Oxford Instruments X-act PentaFET Precision sensor (High Wycombe, UK). The samples were metalized with a Quorum Q150R ES Plus sputter coater (Lewes BN8 6BN, UK) to obtain conductive surfaces.

### 2.3. Bacterial Strains and Culture Conditions

The bacterial strains used in this study were clinical isolates from urinary tract infections, including uropathogenic *Escherichia coli* (UPEC) ATCC 25922, *Klebsiella pneumoniae* ATCC 700603, *Enterococcus faecalis* ATCC 29212, and *Pseudomonas aeruginosa* ATCC 17934. The bacteria were routinely cultured in Luria–Bertani (LB) broth (BD Difco™, Franklin Lakes, NJ, USA), LB agar (BD Difco™, USA), trypticase soy broth (TSB, BD Difco™, USA), and trypticase soy agar (TSA, BD Difco™, USA) for 24–36 h at 37 °C.

### 2.4. In Vitro Evaluation of Antimicrobial Activity

COPESIL^®^ samples were rinsed with 5 mL of sterile 1X phosphate-buffered saline (PBS). Silicone films (negative control) and copper sheets (99% Cu, positive control) of similar diameter to the evaluated films were also included. The films were allowed to dry inside a Petri dish in a laminar flow hood and then sterilized with UV radiation for 15 min on each side (30 min total).

The bacteria were cultured overnight in LB broth at 37 °C. The cultures were then centrifuged at 6000× *g* for 10 min, and the pellets were suspended in sterile PBS. The inoculum was prepared to achieve approximately 1 × 10^7^ colony-forming units (CFUs) in 60 µL, which were then distributed on the surface of each film. After 3 h of incubation, 30 µL of sterile PBS were added to each film to counteract evaporation and prevent dehydration-related bacterial death.

Following contact times of 2, 4, and 6 h, the films were carefully transferred to sterile 50 mL plastic tubes. The bacteria were recovered by adding 5 mL of sterile PBS, followed by vortexing for 30 s, incubation in an ultrasonic bath for 5 min, and another 30 s vortex. Serial dilutions were then performed, and 20 µL of each dilution were plated on MacConkey agar for *E. coli* and *K. pneumoniae*, sheep blood agar for *E. faecalis*, and TSA for *P. aeruginosa*. Plates were incubated for 24 h at 37 °C. All assays were performed in duplicate and repeated at least once.

### 2.5. In Vitro Evaluation of Antibiofilm Activity in a Custom Continuous Flow System

An in-house continuous flow system was designed to evaluate antibiofilm activities. The system consisted of a 1 L bottle for bacterial cultivation connected via tubing to four serially arranged chambers. COPESIL^®^ and control silicone films were placed in these chambers in duplicates. A calibrated pump controlled the culture flow at a rate of 3.3 mL/min, allowing the bacterial suspension to continuously circulate through each chamber, ensuring contact with the films before returning to the cultivation bottle. This cycle was maintained throughout the experiment, conducted at 37 °C for 24 h in a climate-controlled environment.

Prior to the experiment, the films were sterilized under UV light for 30 min, with a total of 15 min per side. The entire flow system, including tubing and chambers, was disinfected with a 10% sodium hypochlorite solution, then thoroughly rinsed with sterile distilled water to eliminate any disinfectant residue. The sterile films were then placed in each chamber under sterile conditions in a laminar flow hood.

Overnight cultures of *E. coli* ATCC 25922 and *K. pneumoniae* ATCC 700603 were grown in LB medium at 37 °C. Following overnight growth, bacterial dilutions were prepared to achieve final concentrations of 1:500 for *E. coli* and 1:10,000 for *K. pneumoniae* in 500 mL of artificial urine medium (AUM), prepared as previously described [46].

After 24 h of incubation, the films were washed with PBS and fixed with 70% methanol for 7 min. Once fixed, the films were air-dried, stained with 0.5% crystal violet for 15 min, and then washed three times with distilled water to remove any unbound stain. The stained films were submerged in 1 mL of 33% acetic acid and vortexed for 20 s to quantify the biofilms. The optical density at 595 nm (OD595) was measured to assess the biofilm mass. Statistical analysis using the Mann–Whitney test was conducted to evaluate significant differences in biofilm formation by *E. coli* and *K. pneumoniae* across the different films. This experiment was performed at least twice in duplicate.

### 2.6. Cellular Cytotoxicity Assay

To evaluate the cytotoxic potential of COPESIL^®^ films, HepG2 cells, a human hepatocellular carcinoma cell line, were cultured in DMEM supplemented with 10% fetal bovine serum (FBS) and 1% antibiotics (penicillin and streptomycin). The cultures were maintained at 37 °C in a humidified atmosphere containing 5% CO_2_.

The cytotoxic effects were quantified using the “In vitro Toxicology Assay Kit, Resazurin Based” (Sigma-Aldrich, Darmstadt, Germany; Cat. No. TOX8-1KT). This assay assesses cell viability by measuring the reduction of resazurin to resorufin, a fluorescent compound, by metabolically active cells. Fluorescence intensity, indicative of cell viability, was quantified using a microplate fluorometer calibrated to excitation and emission wavelengths of 530 and 590 nm, respectively.

HepG2 cells were plated at a density of 5 × 10^5^ cells per well in 6-well plates. Concurrently, four films of each type—silicone control, COPESIL^®^ 1, and COPESIL^®^ 2—were sterilized by UV radiation for 30 min and placed in empty 6-well plates. Each plate then received 2 mL of the culture medium and was incubated for 24 h under standard cell culture conditions.

Following the incubation period, cells were washed three times with 1 mL of PBS and subsequently exposed to the medium conditioned by each film type. Controls included cells exposed to untreated medium and medium incubated with films but without cells. After 24 h of incubation, 200 µL of resazurin reagent was added to each well. The plates were then incubated for another 2 h at 37 °C, after which fluorescence measurements were taken using a Synergy™ HT microplate reader (BioTek Instruments, Winooski, VT, USA).

The recorded fluorescence from the medium incubated with films but without cells was subtracted from each respective sample to correct for non-cellular background fluorescence. The Mann–Whitney test was applied to analyze statistically significant differences in the fluorescence emitted by cells exposed to the different film conditions compared to the controls. Each experimental condition was performed in triplicate.

## 3. Results

### 3.1. Microstructural and Chemical Composition Analysis of COPESIL^®^

**Figure 1:**
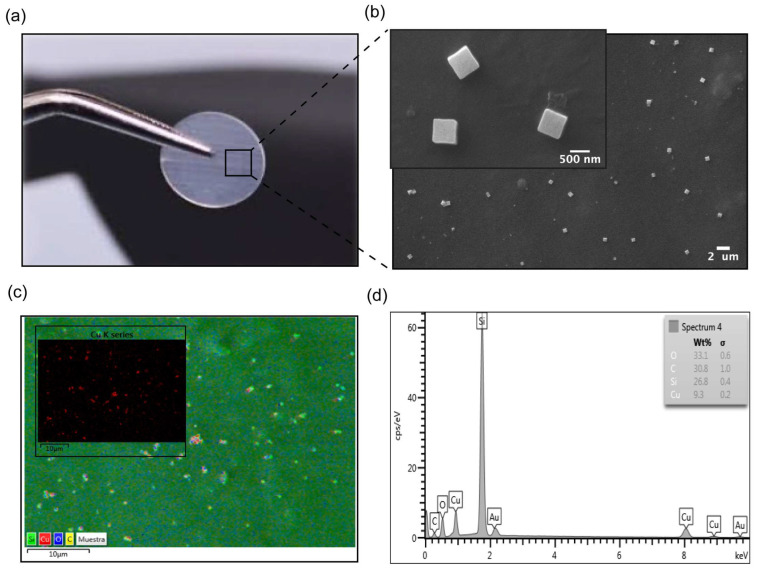
Characterization of COPESIL^®^ films. (**a**) Macroscopic view of a modified silicone film. This image displays a silicone film modified with COPESIL^®^ technology, showing that the film retains its original visual characteristics post-modification, which suggests that the essential physical properties such as color and transparency are preserved. (**b**) SEM micrograph of COPESIL^®^. The micrograph provides a detailed view at the nano-scale, highlighting the uniform distribution of cubic nanostructures, sized approximately 480.48 ± 112.07 nm, across the silicone surface. (**c**) Chemical mapping of COPESIL^®^. This panel illustrates a general chemical map of a representative area where the color intensity varies according to the concentration of different elements, providing insights into the elemental distribution within the material. The inset shows a specific map for copper, with red coloring indicating the presence and uniform distribution of copper nanoparticles. (**d**) EDS spectrum of COPESIL^®^. The EDS spectrum quantifies the elemental composition of the material, highlighting significant peaks for elements like copper, which corroborate the targeted incorporation of antimicrobial agents into the silicone matrix.

The application of COPESIL^®^ technology to silicone films was evaluated through SEM and EDS. These analyses aimed to assess microstructural modifications and determine the elemental composition introduced by the technology. The comprehensive results are depicted in Figure 1, detailing the silicone’s morphology and chemical attributes after modification at the microscopic scale.

Figure 1a demonstrates that the macroscopic visual characteristics, such as the color and transparency of silicone films, remain unchanged post-modification with COPESIL^®^ technology. This observation suggests that the essential physical properties of silicone are preserved while potentially enhancing its functional attributes through microscopic modifications.

The SEM micrograph shown in Figure 1b provides a detailed view of the microstructural changes to the silicone. It reveals the presence of uniformly distributed nanostructures with an average size of 480.48 ± 112.07 nm. This uniform distribution is critical for ensuring consistent performance across the entire material surface.

Figure 1c offers a comprehensive chemical map of a representative area of the COPESIL^®^ material. This map effectively delineates the spatial distribution of various elements within the matrix, with color intensity directly correlating to the concentration levels of each element. The presence of carbon can be observed all over the surface (represented by yellow color), suggesting an adequate surface modification with PEG-Silane monolayers. On the other hand, it is noted that the highest concentration of carbon is in the areas where the nanoparticles are located, evidenced by the overlapping of the red and yellow colors. This finding is consistent with the presence of stabilizing molecules and self-assembled monolayers of PEG and PEG-Silane, since these molecules contain hydrocarbon chains. Such detailed mapping is indispensable for confirming the uniform integration of elements throughout the modified material. Notably, the map highlights the well-distributed, red-colored copper nanoparticles.

**Figure 2:**
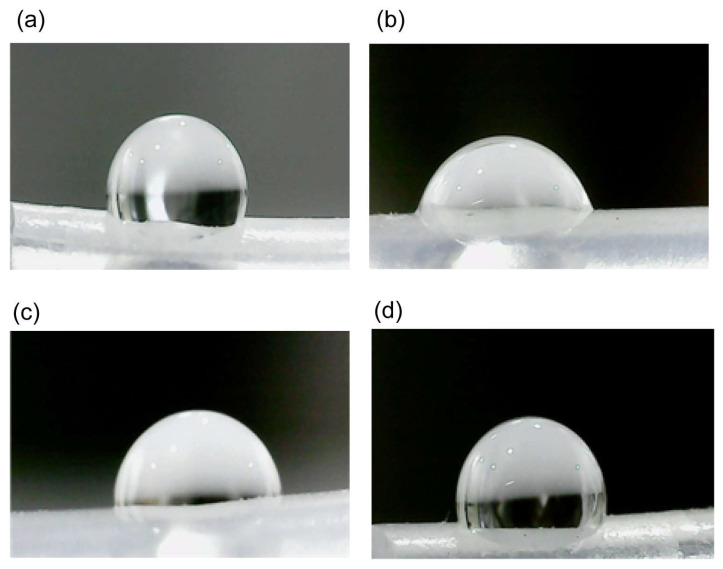
Sequential modifications of the PDMS surface illustrated by water contact angle measurements. (**a**) Baseline. The unmodified PDMS surface shows the original water contact angle. (**b**) Activation. The surface post-HCl treatment demonstrates a reduced contact angle due to the formation of silanol (Si-OH) groups. (**c**) Pegylation. After the incorporation of PEG-Silane, the contact angle slightly increases due to the presence of hydrocarbon chains. (**d**) Functionalization. in the final stage, with copper nanoparticles (CuNPs) embedded, the contact angle slightly increases further, indicating successful immobilization and stabilization of CuNPs on the hydrophobic surface.

Chemical modification of the PDMS surface resulted in a hydrophilic surface. The samples were characterized using the water contact angle (WCA) technique to determine their hydrophilic stability (Figure 2). Static contact angles were measured both before and after modifications. Generally, a decrease in the WCA from 107 ± 1.9° to 72 ± 2.5° was observed following the activation process with HCl, confirming the formation of uniform silanol (Si-OH) groups on the surface. Subsequently, the incorporation of PEG-Silane molecules resulted in an increase in the WCA to 87 ± 3.0, attributed to the presence of hydrocarbon groups. Finally, modification of the silicone films with CuNPs led to a further increase in the WCA to 95 ± 2.7°.

Additionally, Figure 1d provides an EDS spectrum that quantifies the elemental composition of the COPESIL^®^ material. The EDS analysis provided qualitative information on the chemical composition of COPESIL. Characteristic peaks of copper (Kα: 8.0 and Lα: 0.93), oxygen (Kα: 0.53), and silicon (Kα: 1.73) were observed, consistent with the synthesized copper oxide I (Cu_2_O) nanoparticles. The spectrum reveals a significant presence of copper, specifically quantifying its abundance on the material’s surface. This elemental analysis not only supports the uniform distribution seen in the chemical mapping but also underscores the functionalization of silicone with copper. Collectively, these results illustrate the successful application of COPESIL^®^ technology in modifying the surface characteristics of silicone films without altering their fundamental physical properties while simultaneously endowing them with enhanced functional capabilities. This formulation of COPESIL^®^ presented a concentration of 0.123 ± 0.09 pM, as determined by the DLS technique of concentration in solution in the Nano tracking Analisys (NTA).

### 3.2. Antimicrobial Efficacy of COPESIL^®^ Against Clinical Pathogens

We assessed the antimicrobial efficacy of the COPESIL^®^-1 and COPESIL^®^-2 formulations, comparing their performance to standard silicone films (negative control) and copper sheets (positive control). The bacteria used were uropathogenic *Escherichia coli*, *Klebsiella pneumoniae*, *Enterococcus faecalis*, and *Pseudomonas aeruginosa*, commonly implicated in CAUTIs.

Copper sheets, functioning as the positive control, consistently exhibited a reduction in bacterial counts above 99.9% across all strains and at every time point.

COPESIL^®^-1 exhibited strong antibacterial activity, particularly against *E. coli*, reducing bacterial counts by over 81.8% within 2 h of exposure (Table 1). This activity further increased to a reduction of over 99.9% by 6 h. Similarly, the material showed progressive efficacy against *K. pneumoniae*, with bacterial reductions beginning at 76.2% at the 2 h mark and reaching up to 99.8% by 6 h. Its effects against *E. faecalis* and *P. aeruginosa* were more moderate, however, with reductions of 75.3% and 67.8%, respectively, by 6 h.

COPESIL^®^-2 also demonstrated significant antimicrobial activity. It rapidly and consistently demonstrated a >99.9% reduction against *E. coli* from the 2 h time point, sustained throughout the 6 h period (Table 2). The formulation achieved a 97.7% reduction in *K. pneumoniae* counts at 6 h. Moreover, it displayed antimicrobial activity against *E. faecalis* and *P. aeruginosa*, with reductions of 64.5% and 91.3%, respectively, by the end of the 6 h interval.

These results illustrate the antimicrobial properties of the COPESIL^®^ formulations, highlighting their effectiveness, particularly against Gram-negative bacteria such as *E. coli* and *K. pneumoniae*. The observed moderate but important activity against the Gram-positive *E. faecalis* and Gram-negative *P. aeruginosa* suggests a broader spectrum of potential applications for these materials in clinical settings.

### 3.3. Antibiofilm Efficacy of COPESIL^®^

Based on the findings of the *in vitro* antimicrobial activity of COPESIL^®^ formulations against *E. coli* and *K. pneumoniae*, these bacteria were subsequently selected to assess the biofilm inhibitory potential of these materials.

**Figure 3:**
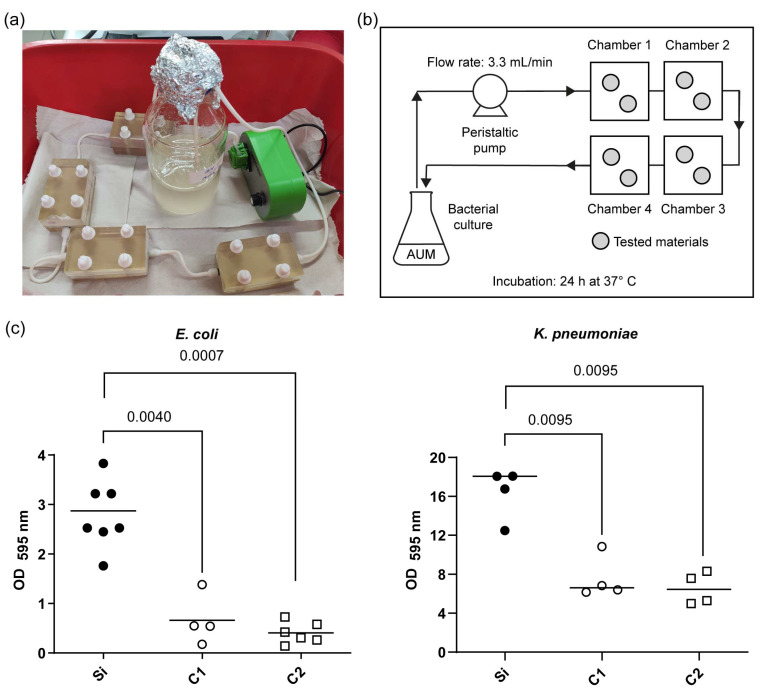
Quantitative analysis of biofilm formation on the COPESIL^®^ and control films. (**a**) Experimental setup showing the continuous flow system used for the biofilm formation assay. (**b**) Schematic drawing of the continuous flow system developed, indicating the flow of artificial urine medium (AUM) through chambers containing the tested materials, with the flow rate set at 3.3 mL/min and an incubation period of 24 h at 37 °C. (**c**) Optical density measurements at 595 nm (OD595) displaying the extent of biofilm formation by *E. coli* (left) and *K. pneumoniae* (right) on various film surfaces after 24 h. The tested films include silicone control films (Si), COPESIL^®^-1 films (C1), and COPESIL^®^-2 films (C2). The data points represent individual measurements, and the horizontal lines indicate the median values. For *E. coli*, biofilms formed significantly less on both COPESIL^®^ films than the silicone control. Similarly, biofilm formation by *K. pneumoniae* was significantly lower on COPESIL^®^ films. Statistical analysis using the Mann–Whitney test shows significant differences, with P-values indicated above the brackets to highlight comparisons between different film types.

Using the custom continuous flow system described in the Materials and Methods section (Figure 3a,b), biofilm assays were conducted in an artificial urine medium to emulate the urinary tract environment where CAUTIs occur.

In this setting, control silicone surfaces, as expected, facilitated substantial biofilm formation, aligning with the known biofilm-forming capabilities of *E. coli* and *K. pneumoniae*. Remarkably, biofilm production by *K. pneumoniae* was observed to be approximately six-fold greater than that of *E. coli.*

In contrast, COPESIL^®^ films exhibited significantly reduced biofilm formation for both bacterial strains compared to the silicone control (Figure 3c). Specifically, COPESIL^®^-1 reduced biofilm formation by 2.7-fold for *K. pneumoniae* and 5.2-fold for *E. coli*. COPESIL^®^-2 demonstrated even more substantial biofilm reductions, achieving 2.8-fold and 7.9-fold decreases for *K. pneumoniae* and *E. coli*, respectively. No statistically significant differences were observed between the biofilm reduction capabilities of the two COPESIL^®^ formulations.

### 3.4. Assessment of Cytotoxicity of COPESIL^®^ in HepG2 Cells

Assessing biocompatibility is paramount in developing biomedical materials such as those incorporating copper nanoparticles like COPESIL^®^ films. This is particularly crucial given the potential for these materials to release copper ions when in contact with bodily tissues, which could pose cytotoxic risks. Using HepG2 cells, which closely mimic human liver metabolism, we evaluated whether the release of copper ions from COPESIL^®^ films adversely affects cellular viability or function.

**Figure 4:**
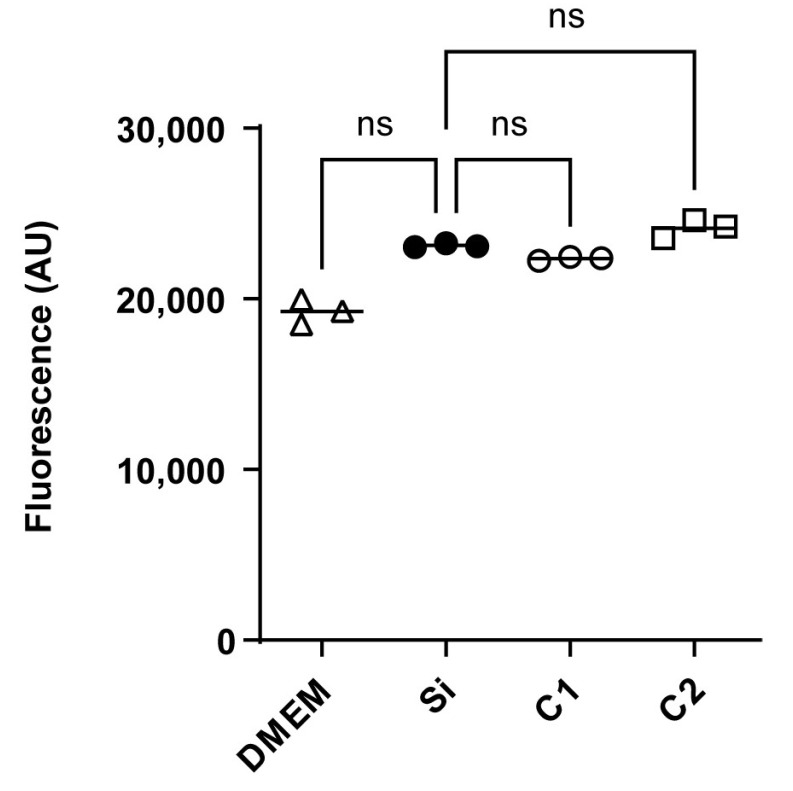
Cytotoxicity assessment in HepG2 cells using resorufin fluorescence. Fluorescence measurements of resorufin, indicative of cellular metabolic activity, are shown for HepG2 cells exposed to different conditions. The fluorescence intensities, measured in arbitrary units (AUs), are shown for cells incubated in DMEM (control) and cells exposed to media conditioned with silicone control films (Si), COPESIL^®^-1 films (C1), and COPESIL^®^-2 films (C2). The data points denote mean fluorescence values obtained from triplicate experiments. The Mann–Whitney test was applied to analyze statistically significant differences. ns = not significant.

Figure 4 shows no substantial variations in cellular metabolism between HepG2 cells subjected to a medium conditioned with COPESIL^®^ films (C1 and C2) and those under control conditions. This observation suggests that COPESIL^®^-1 and COPESIL^®^-2 films do not exhibit cytotoxic effects after 24 h of exposure. Similarly, the silicone control films also displayed no cytotoxicity, underscoring the inert nature of these materials. Moreover, no significant differences in cytotoxicity between the two COPESIL formulations were observed, indicating consistent performance in terms of safety.

These preliminary findings suggest the suitability of COPESIL^®^ films for safe use for applications involving direct contact with human tissues. Despite the presence of copper nanoparticles, the absence of adverse effects on cellular viability and metabolism supports their potential use in various medical devices where biocompatibility is essential. Further studies into longer exposure times and different concentration levels may yield additional insights into the long-term safety and effectiveness of these innovative materials.

## 4. Discussion

The development of COPESIL^®^ technology, as investigated in this study, represents a promising advancement in the field of antimicrobial materials for medical devices, particularly catheters. Integrating PEGylated copper nanoparticles within a PDMS matrix has been shown to preserve silicone’s inherent physical properties and enhance its functionality against microbial colonization and biofilm formation. A second manuscript is being prepared that details, step by step, the modification and physicochemical analysis of the material.

Our findings demonstrate that COPESIL^®^ exhibits antimicrobial activity, particularly against UPEC and *K. pneumoniae*, achieving a reduction in bacterial counts exceeding 99% compared to the silicone control (Table 1 and Table 2). This decrease in bacterial viability is likely due to the well-documented antimicrobial mechanisms of copper nanoparticles, such as membrane disruption, oxidative stress induction, and interference with microbial DNA [26,27,30,31,32,33].

However, the efficacy of COPESIL^®^ against *E. faecalis* and *P. aeruginosa* was less pronounced, which may be linked to the complex copper resistance mechanisms these bacteria possess. The *tcrYAZB* operon in *E. faecalis*, similar to the *copYZAB copper*-homeostasis gene cluster in *Enterococcus hirae*, plays a pivotal role in copper resistance. The presence of this operon allows these bacteria to thrive even in environments with high copper concentrations [47].

In the case of *P. aeruginosa*, copper resistance is even more robust, facilitated by multiple copper export systems intricately linked to its survival and adaptability in hospital environments. A recent review by Virieux-Petit et al. [48] details how copper resistance not only enhances the persistence of this bacterium in hospital water systems but also contributes to its role as a major nosocomial pathogen. In particular, the expression of P1B-type ATPases, like CopA1 and CopA2, and other efflux systems facilitate active copper efflux [49,50], thereby maintaining low intracellular copper levels and enabling resistance to copper-based antimicrobial strategies. Thus, the moderate efficacy of COPESIL^®^ against *E. faecalis* and *P. aeruginosa* highlights the challenges of achieving broad-spectrum antimicrobial activity and underscores the need for further optimization of the nanoparticle formulations and concentrations.

Comparing the antimicrobial performance of COPESIL^®^ with that of other nanoparticle-enhanced surfaces reveals a diverse spectrum of materials and outcomes, particularly involving metals such as silver, zinc, and copper. The study by Desai et al. (2010) [41] highlights the variable efficacy of silver and nitrofurazone in reducing bacterial adherence to urinary catheters. While nitrofurazone showed some effectiveness, silver impregnation had a minimal impact, underscoring the complexity of achieving consistent antimicrobial effects with silver.

In contrast, Sehmi et al. (2015) [38] demonstrated that copper nanoparticles embedded into silicone and polyurethane preserved the materials’ inherent aesthetic properties while exhibiting potent antibacterial activity against pathogens like methicillin-resistant *Staphylococcus aureus* and *E. coli*. This underscores the efficacy of copper in a context similar to that of COPESIL^®^, highlighting its potential for broad clinical application. Further reinforcing the utility of copper, Ballo et al. (2016) [42] investigated silver–copper nanoparticle coatings on catheters and found that these coatings could significantly reduce the incidence of catheter-associated infections.

Additionally, the research by Pulit-Prociak et al. (2020) [35] on poly(vinyl alcohol) (PVA) compositions containing silver, copper, and zinc oxide nanoparticles demonstrated their ability to effectively inhibit the growth of Gram-negative and Gram-positive bacteria. This study highlights the broad-spectrum antimicrobial properties of copper, particularly when used in conjunction with other antimicrobial metals.

Most recently, Ding et al. (2024) [51] reported on the development of copper–phenolic coatings for silicone urinary catheters. These coatings showed remarkable antibacterial properties against both standard pathogens and drug-resistant bacteria, indicating that copper-based coatings can significantly enhance the antimicrobial properties of medical devices, aligning closely with the objectives of the COPESIL^®^ technology. These studies collectively demonstrate that research into antimicrobial biomaterials is an active field, showcasing a variety of innovative approaches and materials aimed at reducing HAIs. Among these, incorporating metal nanoparticles into polymers for medical device applications is a particularly promising strategy.

Our study’s custom continuous flow biofilm model demonstrated that both COPESIL^®^ formulations substantially inhibit biofilm formation against UPEC and *K. pneumoniae* (Figure 3). Similar systems have been reported in the literature and are useful for *in vitro* evaluation of antibiofilm properties [52].

The long-term stability and potential leaching of copper ions from the PDMS matrix warrant further investigation. Developing biomedical materials like COPESIL^®^ necessitates a rigorous evaluation of their biocompatibility to ensure they do not elicit adverse effects on human cells. This is particularly relevant for applications involving direct contact with bodily tissues, where cytotoxicity could compromise cell health and overall treatment efficacy.

HepG2 cells, derived from human liver carcinoma, are frequently employed in cytotoxic evaluations due to their close simulation of human liver metabolic processes [53]. This makes them a common model for assessing the potential toxic effects of new biomaterials intended for medical applications, and indeed, they have been used to evaluate copper toxicity [54]. Initial cytotoxicity assays using HepG2 cells revealed no significant adverse effects, suggesting the initial safety of COPESIL^®^ (Figure 4).

However, one limitation of our study is the *in vitro* nature of the testing environments, which, while insightful, does not capture the complex interactions of a live biological system. The *in vitro* environment lacks the immune system interactions, potential systemic toxicity, and other dynamics present *in vivo*. Future research will involve *in vivo* studies to evaluate the efficacy and safety of COPESIL^®^ and to assess the long-term stability of nanoparticles, their interaction with the immune system, and their overall impact on infection rates and healing outcomes.

Another challenge lies in assessing the material’s behavior under chronic exposure conditions typical of medical implants. Such long-term scenarios are crucial for confirming the material’s safety over extended periods, highlighting the need for a rigorous and sustained assessment to ensure that COPESIL^®^ complies with the stringent safety standards mandated for medical applications.

In conclusion, COPESIL^®^ represents a promising approach to mitigating biofilm-associated risks in medical devices. The next phases of research should focus on optimizing the antimicrobial components for enhanced spectrum efficacy, investigating long-term material stability, and conducting preclinical and clinical trials to validate the *in vitro* successes observed in this study.

## 5. Patents

An application for an international and national patent process has been submitted for the COPESIL^®^ technology. Patent N° WO2023/122846A1.

## Figures and Tables

**Table 1:** Antimicrobial activity of COPESIL^®^-1 during 6 h of contact compared to silicone rubber.

Bacteria	Time (h)	Batch	Number of CFUs Recovered per Sample *		% Reduction Achieved by COPESIL **	% Average Reduction (±SD)
			Silicone	COPESIL^®^-1		
*E. coli* Inoculum: 6 × 10^6^–3 × 10^7^ CFU	2	1	2.25 × 10^7^	1.63 × 10^4^	99.9	81.8 (±25.6)
		2	3.88 × 10^6^	1.41 × 10^6^	63.7	
	4	1	4.50 × 10^6^	4.15 × 10^5^	90.8	95.3 (±6.4)
		2	6.63 × 10^6^	9.75 × 10^3^	99.9	
	6	1	8.25 × 10^6^	<1	>99.9	>99.9
		2	6.75 × 10^6^	<1	>99.9	
*K. pneumoniae* Inoculum: 1 × 10^7^–2 × 10^7^ CFU	2	1	7.50 × 10^6^	1.13 × 10^6^	84.9	76.2 (±12.4)
		2	9.38 × 10^6^	3.06 × 10^6^	67.4	
	4	1	1.13 × 10^7^	9.38 × 10^4^	99.2	81.5 (±25)
		2	5.88 × 10^6^	2.13 × 10^6^	63.8	
	6	1	3.75 × 10^5^	5.31 × 10^3^	99.8	99.8
		2	3.50 × 10^6^	7.50 × 10^2^	99.8	
*E. faecalis* Inoculum: 7 × 10^6^–9 × 10^6^ CFU	2	1	8.75 × 10^6^	1.94 × 10^6^	77.8	59.1 (±6.4)
		2	8.38 × 10^6^	5.00 × 10^6^	40.3	
	4	1	7.50 × 10^6^	3.19 × 10^6^	57.5	56.0 (±6.4)
		2	9.88 × 10^6^	4.50 × 10^6^	54.5	
	6	1	2.13 × 10^6^	7.00 × 10^5^	67.1	75.3 (±11.5)
		2	4.75 × 10^6^	7.88 × 10^5^	83.4	
*P. aeruginosa* Inoculum: 1 × 10^7^–3 × 10^7^ CFU	2	1	2.00 × 10^7^	6.56 × 10^6^	67.2	69.6 (±3.3)
		2	1.26 × 10^7^	3.54 × 10^6^	71.9	
	4	1	2.13 × 10^7^	9.05 × 10^6^	55.4	77.7 (±31.5)
		2	7.25 × 10^6^	1.44 × 10^3^	>99.9	
	6	1	1.63 × 10^7^	9.50 × 10^6^	41.7	67.8 (±36.9)
		2	1.69 × 10^7^	1.02 × 10^6^	94.0	

* Each value corresponds to the average of duplicates of CFUs recovered in two samples evaluated per production batch. ** As compared with silicone rubber.

**Table 2:** Antimicrobial activity of COPESIL^®^-2 during 6 h of contact compared to silicone rubber.

Bacteria	Time (h)	Batch	Number of CFUs Recovered per Sample *		% Reduction Achieved by COPESIL **	% Average Reduction (± SD)
			Silicone	COPESIL^®^-2		
*E. coli* Inoculum: 9 × 10^6^–2 × 10^7^ CFU	2	1	1.60 × 10^6^	<1	>99.9	>99.9
		2	1.50 × 10^6^	<1	>99.9	
	4	1	1.76 × 10^6^	<1	>99.9	>99.9
		2	1.70 × 10^6^	<1	>99.9	
	6	1	1.28 × 10^6^	<1	>99.9	>99.9
		2	1.00 × 10^6^	<1	>99.9	
*K. pneumoniae* Inoculum: 1.2 × 10^7^–1.4 × 10^7^ CFU	2	1	1.21 × 10^7^	1.13 × 10^6^	90.7	69.9 (±29.4)
		2	7.25 × 10^6^	3.69 × 10^6^	49.1	
	4	1	1.41 × 10^7^	9.63 × 10^5^	93.2	87.8 (±7.7)
		2	6.00 × 10^6^	1.06 × 10^6^	82.3	
	6	1	1.16 × 10^7^	2.14 × 10^5^	98.2	97.7 (±0.7)
		2	5.25 × 10^6^	1.47 × 10^5^	97.2	
*E. faecalis* Inoculum: 9.0 × 10^6^–9.1 × 10^6^ CFU	2	1	8.75 × 10^6^	3.69 × 10^6^	57.8	43.9 (±19.7)
		2	7.50 × 10^6^	5.25 × 10^6^	30.0	
	4	1	1.38 × 10^7^	4.00 × 10^6^	71.0	67.8 (±4.6)
		2	1.00 × 10^7^	3.55 × 10^6^	64.5	
	6	1	6.25 × 10^6^	1.00 × 10^6^	84.0	64.5 (±27.5)
		2	5.50 × 10^5^	3.02 × 10^5^	45.1	
*P. aeruginosa* Inoculum: 2.1 × 10^7^–2.9 × 10^7^ CFU	2	1	3.75 × 10^7^	5.88 × 10^6^	84.3	62.7 (±30)
		2	1.59 × 10^7^	9.38 × 10^6^	41.0	
	4	1	3.88 × 10^7^	1.10 × 10^7^	71.6	85.6 (±19.7)
		2	1.78 × 10^7^	7.95 × 10^4^	99.6	
	6	1	5.38 × 10^7^	8.80 × 10^6^	83.6	91.3 (±10.8)
		2	5.63 × 10^5^	6.38 × 10^3^	98.9	

* Each value corresponds to the average of duplicates of CFUs recovered in two samples evaluated per production batch. ** As compared with silicone rubber.

## Data Availability

Data sharing does not apply to this article as no datasets were generated or analyzed during the current study. Materials are not available due to legal restrictions and an ongoing international patent application.

## Abbreviations

The following abbreviations are used in this manuscript:

AUM	Artificial urine medium
CAUTIs	Catheter-associated urinary tract infections
CFUs	Colony-forming units
CRBSIs	Catheter-related bloodstream infections
CuNPs	Copper nanoparticles
EDS	Dispersive spectroscopy
FBS	Fetal bovine serum
HAIs	Healthcare-associated infections
LB	Luria–Bertani
OD_595_	Optical density at 595 nm
PBS	Phosphate-buffered saline
PDMS	Poly(dimethylsiloxane)
PEG	Polyethylene glycol
PU	Polyurethane
PVC	Polyvinylchloride
TSA	Trypticase soy agar
TSB	Trypticase soy broth
UPEC	Uropathogenic *Escherichia coli*
